# Passive Coping Strategies During Repeated Social Defeat Are Associated With Long-Lasting Changes in Sleep in Rats

**DOI:** 10.3389/fnsys.2020.00006

**Published:** 2020-02-19

**Authors:** Laura A. Grafe, Lauren O’Mara, Anna Branch, Jane Dobkin, Sandra Luz, Abigail Vigderman, Aakash Shingala, Leszek Kubin, Richard Ross, Seema Bhatnagar

**Affiliations:** ^1^Department of Psychology, Bryn Mawr College, Bryn Mawr, PA, United States; ^2^Department of Anesthesiology and Critical Care, Children’s Hospital of Philadelphia, Philadelphia, PA, United States; ^3^Department of Biomedical Sciences, School of Veterinary Medicine, University of Pennsylvania, Philadelphia, PA, United States; ^4^Perelman School of Medicine, University of Pennsylvania, Philadelphia, PA, United States; ^5^Behavioral Health Service, Crescenz Veterans Affairs Medical Center, Philadelphia, PA, United States

**Keywords:** stress, PTSD, active coping, SWS, REM, resilience

## Abstract

Exposure to severe stress has immediate and prolonged neuropsychiatric consequences and increases the risk of developing Posttraumatic Stress Disorder (PTSD). Importantly, PTSD develops in only a subset of individuals after exposure to a traumatic event, with the understanding of this selective vulnerability being very limited. Individuals who go on to develop PTSD after a traumatic experience typically demonstrate sleep disturbances including persistent insomnia and recurrent trauma-related nightmares. We previously established a repeated social defeat paradigm in which rats segregate into either passively or actively coping subpopulations, and we found that this distinction correlates with measures of vulnerability or resilience to stress. In this study, we examined differences between these two behavioral phenotypes in sleep changes resulting from repeated social defeat stress. Our data indicate that, compared to control and actively coping rats, passively coping rats have less slow-wave sleep (SWS) for at least 2 weeks after the end of a series of exposures to social defeat. Furthermore, resilient rats show less exaggerated motor activation at awakenings from rapid eye movement (REM) sleep and less fragmentation of REM sleep compared to control and passively coping rats. Together, these data associate a passive coping strategy in response to repeated social defeat stress with persisting sleep disturbances. Conversely, an active coping strategy may be associated with resilience to sleep disturbances. These findings may have both prognostic and therapeutic applications to stress-associated neuropsychiatric disorders, including PTSD.

## Introduction

Posttraumatic stress disorder (PTSD) is a psychiatric disorder that develops following exposure to one or more traumatic events. Its key symptom domains include re-experiencing of the event, avoidance, negative alterations in cognition and mood, and hyperarousal (Armour et al., 2016). It has been argued that the sleep disturbance in PTSD is a major hallmark of the disorder (Ross et al., 1989), entering into the diagnostic criteria twice, once as the phenomenon of re-experiencing the traumatic event during repetitive nightmares and again as insomnia, a manifestation of hyperarousal. Indeed, both a reduced slow-wave sleep (SWS) amount and an increased number of rapid eye movement (REM) sleep interruptions have been described in PTSD (Mellman et al., 2002; Breslau et al., 2004; Habukawa et al., 2007; Kobayashi et al., 2007; Gupta, 2019). Importantly, these disturbances may persist for many years after the traumatic event.

PTSD develops in only a subset of traumatized individuals, whereas others remain resilient. One factor in the vulnerability to the effects of severe stress is the coping strategy adopted to deal with it (Veenema et al., 2003). In different individuals, or under different conditions, either active coping, characterized by the fight or flight response, or passive coping, characterized by immobility and withdrawal, can occur during exposure to stressors (Engel and Schmale, 1972; Koolhaas et al., 1999; Southwick et al., 2005; Wood and Bhatnagar, 2015). The present experiments used an animal model of repeated social defeat stress in which active and passive coping strategies dichotomize into either a resilient or vulnerable trait, respectively, as assessed *post hoc* by various neuroendocrine measures, behavioral tests (Wood et al., 2010; Chen et al., 2015; Wood and Bhatnagar, 2015; Finnell et al., 2017), and markers of inflammation (Pearson-Leary et al., 2017, 2019). Understanding whether such differences in coping strategy are associated with the subsequent development of sleep disturbances akin to those reported in PTSD may help to provide a basis for examining the mechanisms by which traumatic stress affects sleep in vulnerable individuals. An improved understanding of these mechanisms may have both prognostic and therapeutic applications in treating stress-related disorders.

Our goal was to examine whether the coping strategies identified in the repeated social defeat stress model are associated with differences in sleep measures in rats. Specifically, we examined sleep in passively and actively coping rats before, during, and after seven consecutive days of social defeat in adult male rats. Our data suggest that a passive coping strategy in response to repeated social defeat stress leads to long-lasting sleep deficits, including increased time awake and decreased time in SWS. Conversely, an active coping strategy may provide resistance to some disruptions of sleep such as exaggerated motor startle to waking and REM sleep fragmentation.

## Materials and Methods

### Animals

Male Sprague–Dawley rats 65–75 days of age were obtained from Charles River Laboratories (Wilmington, MA, USA). Four cohorts of rats were used in these experiments (36 animals total; groups of: 6, 12, 12, and 6 rats, in that order). They were singly housed under a 12-h light/dark cycle (lights on at 07:00 h and off at 19:00 h) and had food and water available *ad libitum*. Animals were acclimated to the housing and lighting conditions for at least 5 days prior to any experimental procedures. The Institutional Animal Care and Use Committee of The Children’s Hospital of Philadelphia Research Institute approved all experimental procedures.

### Instrumentation Surgery and Telemetric Recordings

All surgical procedures were performed under aseptic conditions. Isoflurane (2–3%) was used to induce and maintain anesthesia throughout the surgery. A telemetric transmitter was implanted through a midline incision into the abdominal cavity and sutured to the peritoneal wall. The transmitter, made of non-reactive silicone elastomer (Physiotel HD-S02; Data Science International, St. Paul, New Brighton, MN, United States), weighs approximately 7 g and occupies a volume of about 3 ml. It has core temperature and motor activity sensors, and two amplifiers for biopotentials. For recording the cortical EEG, two screws were implanted into the skull (1 mm to the right and left of lambda) and connected to one of the amplifiers. For recording the postural muscle electromyogram (EMG), two insulated stainless steel wire electrodes were attached on either side to dorsal neck muscles. After instrumentation, the skin overlying the scalp and the opening into the intraperitoneal cavity were sutured and meloxicam was administered (2 mg/kg, *sc*).

**Figure 1:**
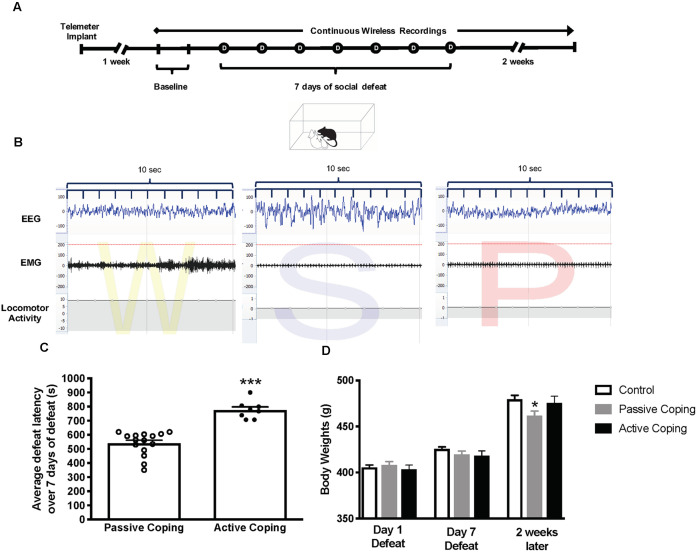
Experimental paradigm, Average defeat latency, and body weights.** (A)** Experimental paradigm depicting the timeline for implantation of telemetry devices, continuous wireless Electroencephalogram (EMG) recordings, and 7-day social defeat exposure. **(B)** Representative EEG, EMG, and activity traces of Wake (denoted as W), slow-wave sleep (denoted as S) and rapid eye movement (REM) sleep (denoted as P for paradoxical sleep). **(C)** Average defeat latency over 7 days of social defeat. Passively coping rats (*n* = 15) have a lower average defeat latency than actively coping rats (*n* = 8; 540 ± 22 s vs. 776 ± 22 s, respectively). **(D)** Body weights for control (*n* = 7), passively (*n* = 15), and actively (*n* = 8) coping rats on the first day of defeat, last day of defeat, and 2 weeks later. Groups do not differ in body weight on day 1 or day 7 of defeat, but passively coping rats weigh significantly less than control or actively coping rats 2 weeks after defeats have ended. *P < 0.05, ***P < 0.001.

After 1 week of recovery from surgery, continuous wireless recordings were initiated by remote activation of a magnetic switch in the transmitter and continued for 24 h a day for 23 days. In each animal, the first 2 days of the protocol were used to collect baseline activity. Then, from days 3 through 9, each animal was exposed to the standardized social defeat paradigm (except for control rats), and recording was continued for 2 weeks after the last defeat day; Figure 1A).

### Social Defeat Paradigm

The social defeat paradigm used in this study was based on the resident-intruder model originally employed by Miczek (1979). Social defeat exposure was applied within 2 h of lights on (starting at approximately 09:00 h each day). Control rats were not exposed to this defeat paradigm but rather placed in novel cages with a divider for 30 min while other rats underwent the defeat procedures. It should be noted that this novel cage stress has been shown to affect measures of sleep in previous studies (Cano et al., 2008). During social defeat, one of the experimental rats (intruder) was placed once each day for seven consecutive days into the home cage of an unfamiliar Long Evans retired breeder (resident). Characteristically, the resident and intruder investigate each other for a short period (1–3 min); this is followed by an attack by the resident, ending with the defeat of the intruder. A defeat is pronounced when the intruder displays a supine posture and freezes for 2–3 s. The latency to the defeat is then recorded. The resident and intruder are then separated by a wire mesh barrier until 30 min elapses from the time of initial placement of the intruder into the resident’s cage. The barrier allows for visual, auditory, and olfactory contact but prevents any further physical contact. As a way to minimize risk for excessive injury, if no defeat occurs within 15 min (900 s), the rats are separated with a wire mesh barrier for the remaining 15 min; in this case, the defeat latency is entered as 900 s. Once the 30-min social stress procedure has concluded, the experimental rat is returned to its home cage. The average defeat latency for each rat over the course of 7 days was then calculated and entered into an R script used to perform cluster analyses on average defeat latencies (code available at www.github.com/cookpa/socialdefeat) as described in Grafe et al. (2018). Briefly, the bootstrap classification starts from the assumption that the average latencies are drawn from a bi-modal distribution. Latencies that are reliably classified as active coping have probability 1.0, and those classified consistently as passive coping have probability 0.0. Some rats with a value between 0.1 and 0.9 changed their classification in more than 10% of the bootstrap samples; in the present study, three animals were excluded based on this criterion. Many more animals were classified as passive copers than active copers. In past work, using this analysis has yielded similar segregation of groups (Chen et al., 2015; Pearson-Leary et al., 2017, 2019; Grafe et al., 2018). Moreover, three additional animals had unreadable telemeter recordings (see next section). Ultimately, the numbers per group were as follows: Controls (7), Passive Coping (15), Active Coping (8).

### Sleep Scoring and Analysis

**Figure 2:**
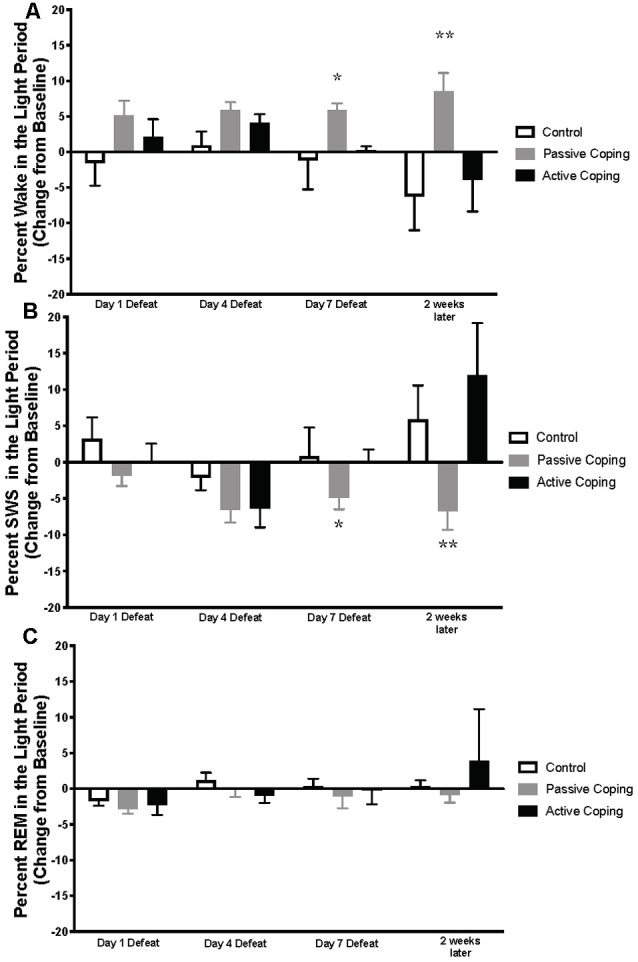
Percent wake, SWS, and REM in the light period presented as change from baseline on Day 1, 4, and 7 of Defeat, and 2 weeks later. **(A)** Percent time spent awake during the light period in control (*n* = 7), passively (*n* = 15), and actively (*n* = 8) coping rats. Passively coping rats spend more time awake than control and actively coping rats after 7 days of defeat and this persists 2 weeks after defeat. **(B)** Percent time spent in slow-wave sleep (SWS) during the light period in control (*n* = 7), passively (*n* = 15), and actively (*n* = 8) coping rats. Passively coping rats displayed significantly less time in SWS compared to the other two groups after 7 days of defeat, and this persisted 2 weeks after defeat. **(C)** Percent time spent in REM sleep during the light period in control (*n* = 7), passively (*n* = 15), and actively (*n* = 8) coping rats. No significant differences in REM sleep were detected between groups at any time point during the light period. *P < 0.05, **P < 0.01.

**Figure 3:**
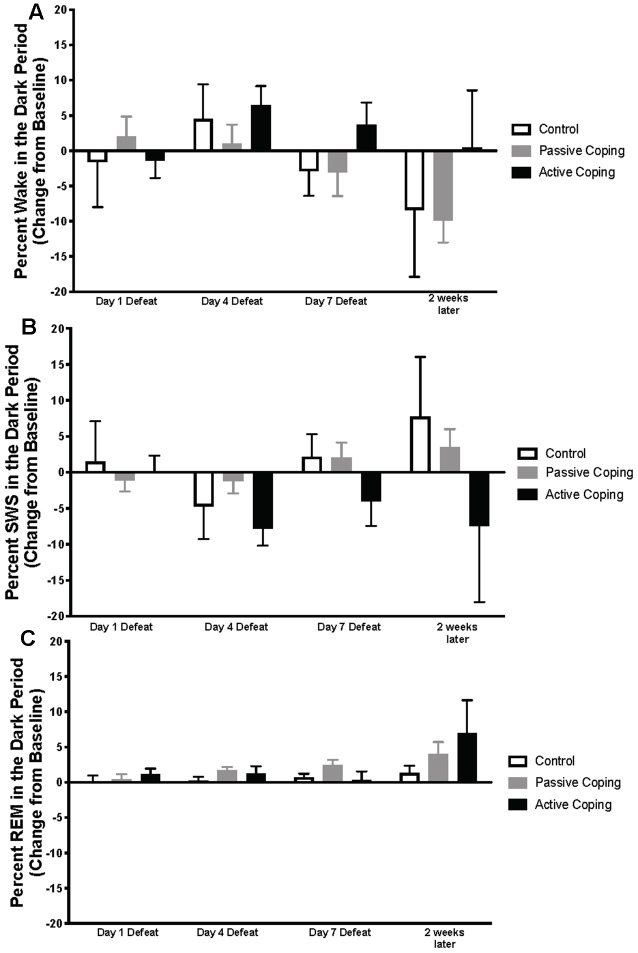
Percent wake, SWS, and REM in the dark period presented as change from baseline on Day 1, 4, and 7 of Defeat, and 2 weeks later. **(A)** Percent time spent awake during the dark period in control (*n* = 7), passively (*n* = 15), and actively (*n* = 8) coping rats. Groups did not significantly differ in the percent of time they spent in wake at any time point. **(B)** Percent time spent in slow-wave sleep (SWS) during the dark period in control (*n* = 7), passively (*n* = 15), and actively (*n* = 8) coping rats. Groups did not significantly differ in the percent of time they spent in SWS at any time point. **(C)** Percent time spent in REM sleep during the dark period in control (*n* = 7), passively (*n* = 15), and actively (*n* = 8) coping rats. Groups did not significantly differ in the percent of time they spent in REM at any time point.

Neuroscore software (Data Science International, St. Paul, New Brighton, MN, United States) was used for sleep scoring in 10 s epochs. Wake and sleep percentage data were calculated using the automated Rodent Sleep Scoring Module 2 in Neuroscore. Neuroscore uses a probability matrix to determine the most likely wake or sleep state. The general states scored are: wake (denoted as W on traces), slow-wave sleep (denoted as S on traces) and REM sleep (denoted as P on traces, for paradoxical sleep); For example traces of each state, see Figure 1B. To determine each state, three signals are used: EEG, EMG and Activity. The EEG data is analyzed by Fast Fourier Transform (FFT), an algorithm that calculates the discrete Fourier transform (DFT) of a sequence, or its inverse (IDFT). A Fourier analysis converts a signal from its time-domain (in the case of EEG) to a representation in the frequency domain. The two bands of interest examined in EEG are Delta power (default 0.05–4 Hz) and the Theta power (default 4–8 Hz). EMG signals are analyzed by amplitude in relation to the baseline. Activity data are derived from an algorithm that compares the signal strength between the device and the receiver with which it is communicating. There are natural fluctuations that occur when the animal is moving, and from those fluctuations, the algorithm generates an activity measurement in counts. For more information, see https://support.datasci.com/hc/en-us/articles/115005030328-Understanding-the-Ponemah-Activity-Derived-Parameter. Depending the value of EMG and presence of activity (meaning transmitter is moving), the epoch will likely be scored as awake. If EMG is low and there is no activity then the Delta Ratio (Delta power dived by total power from 0.5 Hz to 25 Hz) and the Theta Ratio (Theta power divided by Delta power) is examined. If there is high Delta power the epoch is likely scored as SWS, and if there is high Theta power, the epoch is likely scored as REM. As long as the signals are relatively clean, this algorithm is very reliable. The accuracy of the recognition of sleep-wake stages was visually cross-checked by two separate research assistants blind to group conditions. Percentage time in wake and sleep states (wake, slow-wave sleep, REM sleep), number of occurrences of total awakenings vs. exaggerated motor activation at awakening, as well as REM sleep continuity were analyzed. The data were analyzed and averaged across the two baseline days; then for days 1, 4, and 7 of defeat; and then for 1 day 2 weeks later. Sleep stage percentages were split into light and dark periods and presented as change relative to baseline. In the light period (Figure 2), data were analyzed from 10 am to 7 pm on the days of defeat (in order to capture the light period after the end of defeat until lights-off). The same time frame (10 am–7 pm) was analyzed for the baseline and the “2 weeks later” time points. For the dark period (Figure 3), data were analyzed from 7 pm to 6 am (starting at lights-off) at the same time points as in the lights-on analyses.

**Figure 4:**
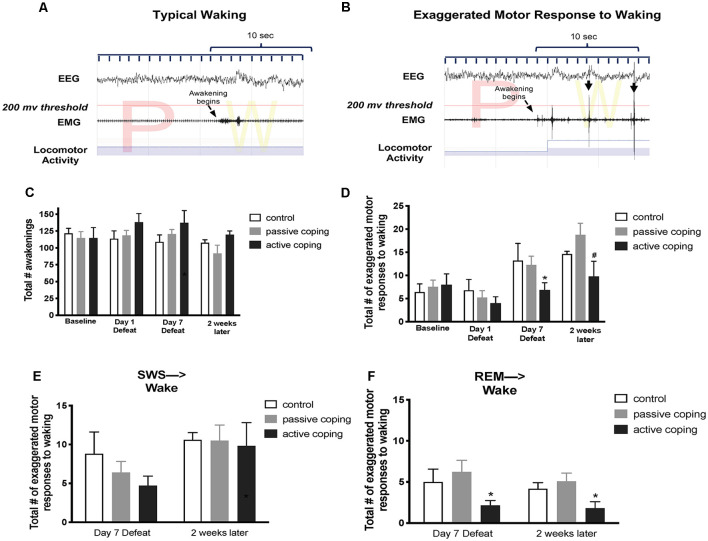
Number of exaggerated motor responses during transitions to waking from SWS or REM sleep in the light period at baseline, Day 1 of defeat, Day 7 of defeat, and 2 weeks after the last defeat.** (A)** Representative typical awakening: a trace of EEG, EMG, and locomotor activity data during arousal from a REM sleep episode (initiated at arrow). Note that EMG spikes do not go above the 200 μV threshold in red within 10 s of the sleep transition. **(B)** Representative exaggerated motor response at awakening: a trace of EEG, EMG, and locomotor activity data during arousal from a REM sleep episode (initiated at arrow). Note the recurrent EMG spikes greater than 200 μV (denoted with black arrows) that occur within 10 s of the sleep transition with concurrent locomotor activity. **(C)** Total counts of awakenings during 6 h of the light period at baseline, defeat day 1, defeat day 7, and 2 weeks after the last defeat exposure. No significant differences in the total number of awakenings were detected between groups at any time point. **(D)** Total counts of exaggerated motor responses to waking during 6 h of the light period at baseline, defeat day 1, defeat day 7, and 2 weeks after the last defeat exposure. Actively coping rats (*n* = 8) displayed less exaggerated motor responses to waking than both control (*n* = 7) and passively coping rats (*n* = 15) after day 7 of defeat, and this persisted 2 weeks later.** (E,F)** Exaggerated motor responses counted separately for arousals from SWS and REM sleep, respectively. Compared to control (*n* = 7) and passively coping rats (*n* = 15), actively coping rats (*n* = 8) had significantly fewer exaggerated motor responses to waking from REM sleep specifically but not from SWS. *P < 0.05, ^#^*P* < 0.1.

Total awakenings over a 6-h period (10 am–4 pm) were counted by neuro score software on defeat days 1 and 7; and 2 weeks after the last defeat. Within those same time points, exaggerated motor responses to waking were identified by visual examination of the nuchal EMG and locomotor activity over a 10 s period following the termination of each sleep bout. Awakenings were categorized as exaggerated when the burst of nuchal EMG crossed a pre-set threshold (200 μV), and there was concurrent locomotor activity, within the first 10 s after awakening. The EMG threshold used in this analysis was validated in a separate subset of rats in which a systematic search was conducted for a threshold value that effectively discriminated between awakenings with and without immediate transition into a motor response. Figures 4A,B show example traces of a typical awakening and an exaggerated motor response to waking, respectively.

Fragmentation of REM sleep was assessed by visually scoring the relative times of occurrence of individual REM sleep episodes. According to the classification introduced by Amici et al. (1994), two types of REM sleep episodes were distinguished: those that are followed by another REM sleep episode after an interval longer than 3 min (“single” episodes, sin-REM), and those followed by another REM sleep episode after an interval of 3 min or less (“sequential” episodes, seq-REM). The total numbers of episodes of each type were counted over 6 h of recording (10 am–4 pm) at baseline; on defeat days 1, 4 and 7; and 2 weeks after the last defeat. Seq-REM episodes are, on average, of shorter duration than sin-REM episodes. Also, seq-REM episodes tend to occur in clusters (Amici et al., 1994). Importantly, a shift toward more seq-REM was previously shown to be indicative of increased fragmentation of REM sleep (Amici et al., 1994; DaSilva et al., 2011).

### Statistical Analysis

Data are presented as the mean ± standard error of the mean (SE). For comparison of defeat latencies between the actively coping and passively coping groups, as validated in our previous studies (Wood et al., 2010; Chen et al., 2015; Pearson-Leary et al., 2017, 2019; Grafe et al., 2018), we used a Student’s *t*-test. For analysis of all other measures, a two-way repeated-measures ANOVA (Group × Time before/during/after defeat exposure) was used followed by Tukey’s *post hoc* *t*-tests (GraphPad Prism; GraphPad Software, La Jolla, CA, USA). In all analyses *α* = 0.05 was regarded as indicative of statistical significance.

## Results

Animals exposed to repeated social defeat were categorized as either passively coping or actively coping rats based on their average latency to defeat over 7 days (Figure 1C). The average latencies for the seven defeat days were 540 ± 22 s vs. 776 ± 22 s (*p* < 0.0001) in the passively coping and actively coping groups, respectively. Body weights for the control and the passively and actively coping rats were recorded on the first and last days of defeat, and 2 weeks later (Figure 1D). A two-way ANOVA revealed a main effect of day and an interaction between day and group (Day, *F*_(2,54)_ = 675.2, *p* < 0.0001; Day × Group, *F*_(4,54)_ = 8.0, *p* < 0.0001). *Post hoc* tests revealed that the groups did not differ from each other in body weight on day 1 or day 7 of defeat, but passively coping rats weighed significantly less than both control and actively coping rats 2 weeks after defeats had ended. Thus, the coping strategy used during repeated social defeat had a long-term effect on body weight.

Percentages of time in wake and different sleep stages during the light period were calculated and presented as change from baseline on Days 1, 4, and 7 of defeat, and 2 weeks later in the control and passively and actively coping groups (Figure 2; For raw percentage data including baseline recordings, please see Supplementary Figure S1). A 2-way ANOVA revealed a significant main effect of group for percent time in awake (wake time/total recording period time; Figure 2A; Group, *F*_(2,22)_ = 8.5, *p* < 0.01). *Post hoc* tests revealed that, compared to control and actively coping rats, passively coping rats had a higher wake percent after 7 days of defeat and this persisted 2 weeks after defeat. For SWS, a 2-way ANOVA revealed main effects of time and group, and an interaction between time and group (Figure 2B; Time, *F*_(2,37)_ = 6.3, *p* = 0.006; Group, *F*_(2,23)_ = 5.6, *p* = 0.01; Time × Group, *F*_(6,62)_ = 2.8, *p* = 0.02). Specifically, *post hoc* tests indicated that by day 7 of defeat, the passively coping group displayed a significantly lower SWS percent compared to the other two groups, and this persisted 2 weeks after the last defeat. Finally, no significant differences in REM sleep percent (REM sleep time/total recording period time; Figure 2C) were detected between groups at any time point during the light period. In sum, after repeated social defeat, passively coping rats showed a distinct decrease in SWS and an increase in wake during the light period (when sleep should be prominent) compared with control and actively coping rats.

Percentages of time in wake and different sleep stages during the dark period were also calculated and presented as change from baseline on Days 1, 4, and 7 of defeat, and 2 weeks later in the control and passively and actively coping groups (Figure 3; For raw percentage data including baseline recordings, please see Supplementary Figure S2). In contrast to the group differences observed in the light period, no significant differences in the wake, SWS, or REM were detected during the dark period.

In the analysis of sleep to wake transitions, we counted the total awakenings as well as the episodes of exaggerated motor responses to waking during the light period. No significant differences in the total number of awakenings were detected between groups at any time point (Figure 4C). A 2-way ANOVA revealed a main effect of time on the total number of exaggerated motor activations at waking (Figure 4D; *F*_(3,45)_ = 11.0, *p* < 0.0001). Further analysis revealed that, compared to both control and passively coping rats, actively coping rats displayed fewer exaggerated motor activations to waking on day 7 of defeat, and this persisted 2 weeks later. We then separately counted episodes of exaggerated motor activation at awakening from SWS and REM sleep. No group-related differences were present on any day for SWS (Figure 4E). In contrast, for REM sleep, actively coping rats showed significantly fewer exaggerated responses compared with control or passively coping rats on defeat day 7, and these effects persisted at the 2-week time point (*p* = 0.0476 and *p* = 0.0428, respectively; Figures 4E,F). Thus, rats coping actively during defeat exhibited lower exaggerated motor responses at awakening on the 7th day of defeat, these lower numbers persisted 2 weeks post-stress and were due to awakenings from REM rather than awakenings from episodes of SWS.

As coping strategy during social defeat stress affected arousal from REM sleep, we also investigated REM sleep continuity by examining REM sleep microarchitecture (Table 1). As described in the “Materials and Methods” section, single and sequential REM sleep (sin-REM and seq-REM) episodes were counted. The number of sin-REM sleep episodes did not significantly differ among groups at any time point. In contrast, a 2-way ANOVA revealed a main effect of time (*F*_(3,61)_ = 4.4, *P* < 0.01) for seq-REM sleep episodes. Further analysis indicated that, compared to control and passively coping rats, actively coping rats had significantly fewer seq-REM sleep episodes on defeat day 4.

**Table 1:** Continuity of REM sleep (single vs. sequential episodes of REM) in the light period at baseline, Day 1 Defeat, Day 4 Defeat, Day 7 Defeat, and 2 weeks later.

	Number of Single REM Episodes			Number of Sequential REM Episodes		
	Control	Passive	Active	Control	Passive	Active
Baseline	15.67 ± 1.3	16.50 ± 1.4	13.17 ± 2.2	9.50 ± 1.8	9.08 ± 0.9	8.33 ± 1.9
Day 1 Defeat	9.83 ± 2.2	13.75 ± 1.0	13.33 ± 2.2	11.50 ± 1.6	11.08 ± 1.3	10.50 ± 1.8
Day 4 Defeat	13.00 ± 1.9	10.42 ± 0.8	12.00 ± 1.7	12.00 ± 0.9	13.08 ± 0.9	8.67 ± 1.0*
Day 7 Defeat	13.33 ± 2.5	11.92 ± 1.1	13.17 ± 1.4	8.67 ± 1.8	8.92 ± 0.9	10.50 ± 0.6
2 Weeks Later	14.00 ± 1.9	14.25 ± 1.5	13.67 ± 2.7	7.67 ± 1.1	5.75 ± 1.6	7.00 ± 2.3

The number of single REM sleep episodes did not significantly differ between groups at any time point. For the sequential REM sleep episodes (those with an inter-REM episode of <3 min), actively coping rats had significantly less sequential REM sleep episodes than control or passively coping rats after Day 4 of defeat. *P < 0.05.

## Discussion

These experiments are the first to examine individual differences in sleep amount and structure in relation to the coping strategy following exposure to social defeat. Specifically, we used a social defeat paradigm that generates two different populations of rats that demonstrate either a passive or an active coping strategy, based on the average latency to defeat. These distinct strategies previously have been related to vulnerability or resilience to stress, respectively (Wood et al., 2010; Chen et al., 2015; Pearson-Leary et al., 2017, 2019). Notably, passively coping rats exhibit increased immobility in the forced swim test and reduced social interaction, indicative of increased helplessness and anxiogenic behavior, respectively. We hypothesized that actively coping (resilient) and passively coping (vulnerable) rats would exhibit differences in sleep architecture during exposure to social defeat, and that these differences would again be demonstrated 2 weeks after the last defeat session. We found that, compared to actively coping rats, passively coping rats had a higher wake percent and a lower SWS percent during, and 2 weeks following, social defeat. In addition, actively coping rats demonstrate resilience to particular sleep deficits such as exaggerated motor responses during spontaneous awakenings and reduced fragmentation of REM sleep episodes after social defeat. Together, these data suggest that a passive, compared to an active, coping strategy during and following repeated social defeat stress is associated with long-lasting sleep changes that can be construed as impairments.

We found that a single exposure to social defeat did not reduce SWS, consistent with other studies (Meerlo et al., 1997, 2001). We also did not see significant differences in SWS with 4 days of defeat. However, after 7 days of defeat, passively coping rats displayed significantly reduced SWS. This time dependence was expected because coping strategy becomes fully established after the fourth defeat (Wood et al., 2010). Among the highly variable polysomnographic findings in PTSD, decreased SWS is one of the most consistent abnormalities across studies (Kobayashi et al., 2007; Richards et al., 2013; Pace-Schott et al., 2015). Thus, our findings showing decreased slow-wave sleep after the use of a passive coping strategy in response to social defeat may have clinical relevance.

A recent study examined how confrontational behaviors during a single social defeat affected sleep (Kinn Rød et al., 2014). Although defeated rats, compared with controls, did not show changes in SWS, a subgroup demonstrating more fighting behaviors exhibited fragmented SWS. Hence, in this short term model of social defeat, an active coping strategy may not be beneficial, but with additional defeat experiences, as in the current paradigm, active coping behavior may be associated with resilience to the effects of defeat on sleep.

In parallel to the reduction in time spent in SWS, passively coping rats also displayed increased time in wake after 7 days of defeat, and this persisted 2 weeks after the end of social defeat. Insomnia is prominent in individuals exposed to trauma, including those who go on to develop PTSD (Lavie, 2001; Mellman and Hipolito, 2006). Thus, the increase in wake we observed in the light period, rats’ inactive time, may have a clinical counterpart.

Recurrent distressing dreams are a very common reexperiencing symptom of PTSD (Ross et al., 1989; Mellman et al., 1995; Mellman and Hipolito, 2006; Germain et al., 2008; Germain, 2013). Such dreams often lead to awakening, in which case they are defined as nightmares. Nightmares are characterized by autonomic arousal, and the nightmares that occur in PTSD, which may emerge from non-REM sleep, as well as REM sleep, is associated with leg movements and/or respiratory events (Phelps et al., 2018). Nightmares are highly deleterious in PTSD, reducing the quality of life (El-Solh, 2018). Furthermore, treatments targeted to nightmares significantly improve the overall quality of life (Calegaro et al., 2019) Therefore, we investigated the characteristics of awakenings from sleep in our social defeat model by analyzing motor activation accompanying their spontaneous awakenings from sleep. Actively coping rats did not have as many exaggerated arousals from REM sleep as passively coping or control rats and this was apparent 2 weeks after defeats ended. Thus, an active coping strategy in response to repeated stress may protect against a phenotype similar to nightmares as observed in PTSD.

As coping strategy during social defeat stress specifically affected awakenings from REM sleep, we further analyzed the continuity of REM sleep. In PTSD patients, the quality of REM sleep appears to be altered. Notably, studies have found that the latency of REM onset is delayed and REM episodes are fragmented (Mellman et al., 2014; Yehuda et al., 2015) and these are considered detriments in REM sleep initiation and maintenance. We found that social defeat stress did not affect latency to REM sleep (data not shown), thus it did not affect REM sleep initiation. We assessed REM sleep maintenance by classifying single and sequential REM episodes (Sin-REM and Seq-REM, respectively; Amici et al., 1994). A sin-REM episode is on average, longer, than a seq-REM episode, and seq-REM episodes tend to occur in clusters (Amici et al., 1994). Importantly, a shift toward more seq-REM was previously shown to be indicative of increased fragmentation of REM sleep (Amici et al., 1994; DaSilva et al., 2011).

Our data indicate that actively coping rats were resilient to detriments in REM sleep maintenance compared to passively coping rats. Specifically, actively coping rats had less seq-REM episodes (fragmentation) after 4 days of defeat, when coping strategies have just begun to establish (Wood et al., 2010). However, this effect did not persist through the remainder of the defeat and post-defeat period. Thus, coping strategy is not associated with long-lasting changes in REM fragmentation after social defeat.

The changes in sleep observed in our studies were present 2 weeks after the end of a defeat. Hence, these effects are long-lasting. This has particular relevance for psychiatric disorders such as PTSD, in which symptoms are often chronic, albeit subject to waxing and waning. Undeniably, these long-lasting alterations in sleep must be mediated by neurobiological changes.

Recent studies have begun to uncover some possible neural substrates underlying stress-induced sleep changes. For example, one report indicates that a kappa-opioid receptor antagonist reversed long-lasting social defeat stress-induced sleep deficits (Wells et al., 2017). Another possible substrate of sleep changes after social defeat is the orexins, neuropeptides that are important in maintenance of wakefulness. We have previously shown that actively coping rats have reduced orexin mRNA compared to control and passively coping rats (Grafe et al., 2018). These reductions might protect actively coping rats from the impact of social defeat stress on sleep amount and continuity and, in particular, reduce the number of transitions from REM sleep to wakefulness. Lastly, a very recent study has uncovered a peripheral substrate that may predict sleep changes after stress (brain-derived neurotrophic factor, BDNF; Sweeten et al., 2020). Future studies are required to determine both the neural and peripheral substrates that underlie the enduring disruption in sleep observed in passively coping rats.

In sum, a passive coping strategy in response to repeated social defeat increases the percent time spent awake and reduces the percent time spent in SWS compared to control and actively coping rats, deficits similar to those observed in PTSD. The slow-wave activity generated during SWS is thought to facilitate the reorganization of cortical circuitry supporting cognition (Wilckens et al., 2018). Thus, it is possible that a passive coping strategy during repeated social defeat may lead to cognitive impairment, a phenomenon frequently observed in many stress-related disorders including PTSD (Vasterling et al., 1998). In addition, actively coping rats show fewer exaggerated motor activations during spontaneous awakenings from REM sleep, a phenotype comparable to the occurrence of nightmares in PTSD (Mellman and Hipolito, 2006). Actively coping rats also show less REM sleep fragmentation, a measure of REM discontinuity, compared with control and passively coping rats during the light period, but this effect is not long-lasting. Overall, the results presented here demonstrate that coping strategy is an important determining factor in the development of sleep deficits associated with repeated stress. Taken together with our previous work, individuals that passively cope with social defeat exhibit sleep deficits accompanied by increases in anxiety-like behaviors and pro-depressive helplessness behaviors. Accordingly, a coping strategy may also be an important determinant of sleep deficits in stressed humans. Future studies should determine whether treatments that reduce anxiety-related behaviors can also reduce these sleep deficits in passively coping individuals.

## Data Availability Statement

The datasets generated for this study are available on request to the corresponding author.

## Ethics Statement

The animal study was reviewed and approved by The Institutional Animal Care and Use Committee of The Children’s Hospital of Philadelphia Research Institute (CHOP IACUC).

## Author Contributions

LG collected data, analyzed the data, and wrote the manuscript. LO’M, AB, JD, and AS analyzed the data. SL and AV contributed to data collection. LK, RR, and SB contributed to experimental design and analysis of data. LG, LK, RR, and SB edited the manuscript.

## Conflict of Interest

The authors declare that the research was conducted in the absence of any commercial or financial relationships that could be construed as a potential conflict of interest.

## Editor’s Note

Arun Asok edited the article in collaboration with Eric R. Kandel, Columbia University, United States
