# Erratum: Passive Coping Strategies During Repeated Social Defeat Are Associated With Long-Lasting Changes in Sleep in Rats

**DOI:** 10.3389/fnsys.2021.782603

**Published:** 2021-10-05

**Authors:** Frontiers Production Office

**Affiliations:** Frontiers Media SA, Lausanne, Switzerland

**Keywords:** stress, PTSD, active coping, SWS, REM, resilience

Due to a production error, the Editor's note was included in the Editor's affiliation. The publisher apologizes for this mistake.

The original article has been updated.

