# Supplementary material for: Passive Coping Strategies During Repeated Social Defeat Are Associated With Long-Lasting Changes in Sleep in Rats
Source: Front Syst Neurosci. 2020 Feb 19;14:6. doi: 10.3389/fnsys.2020.00006 (PMC7043017; doi:10.3389/fnsys.2020.00006)
Supplement: Supplementary file 1 [file Data_Sheet_1.docx]

**Supplemental Figure Results**

Percentages of time in wake and different sleep stages during the light period were calculated at baseline, on Days 1, 4, and 7 of defeat, and two weeks later in the control and passively and actively coping groups (**Supplemental Figure 1**). A 2-way ANOVA revealed a significant interaction between time and group for percent time in wake (wake time/total recording period time; **Supplemental Figure 1A;** Time, (F(4,90) = 1.9, P = 0.1; Time x Group, F (8,90) = 2.5, p < 0.05). Post hoc tests revealed that, compared to control and actively coping rats, passively coping rats had a higher wake percent two weeks after defeat. For SWS, a 2-way ANOVA revealed main effects of time and group, and an interaction between time and group (**Supplemental Figure 1B**; Time, F (4, 56) = 4.8, p = 0.007; Group, F (2, 24) = 11.4, p = 0.0003; Time x Group, F (8, 88) = 2.7, p = 0.01). Specifically, post hoc tests indicated that these groups did not differ at baseline, but both defeat groups had a decreased SWS percent (SWS time/total recording period time) on days 1 and 4 of defeat. By day 7 of defeat, the passively coping group displayed a significantly lower SWS percent compared to the other two groups, and this persisted two weeks after the last defeat. Finally, a 2-way ANOVA revealed a main effect of group on REM sleep percent (REM sleep time/total recording period time; **Supplemental Figure 1C**, F(2,24 = 11.9, p <0.001). Post hoc tests indicated that, compared to the control group, both defeat groups had a higher REM sleep percent at all time points, including baseline. On the other hand, coping strategy did not significantly affect REM sleep percent in the light period. In sum, after repeated social defeat, passively coping rats showed a distinct decrease in SWS and an increase in wake during the light period (when sleep should be prominent) compared with control and actively coping rats.

Percentages of time in wake and different sleep stages during the dark period were also calculated at baseline, on Days 1, 4, and 7 of defeat, and 2 weeks later in the control and passively and actively coping groups (**Supplemental Figure 2**). In contrast to the group differences observed in the light period, no significant differences in wake or SWS were detected during the dark period. However, a 2-way ANOVA revealed main effects of both time and group on percent time in REM sleep ( F (4,114) = 5.7, p <0.001 and F (2, 84) = 21.0, p <0.001, respectively). Post hoc tests indicated that both defeat groups displayed a significantly higher REM sleep percent than the control group at every time point, similar to the finding in the light period.

**Supplemental Figure Legends**

**Figure 1. Percent Wake, SWS, and REM in the light period at Baseline, Day 1, 4, and 7 of Defeat, and 2 weeks later**

**A:** Percent time spent awake during the light period in control (n = 7), passively (n = 15), and actively (n =8) coping rats. Passively coping rats spend more time awake than control and actively coping rats two weeks after defeat. **B:** Percent time spent in slow wave sleep (SWS) during the light period in control (n = 7), passively (n = 15), and actively (n = 8) coping rats. Defeated rats spent significantly less time in SWS on Day 1 and 4 of defeat. By 7 days of defeat, only the passively coping rats displayed significantly less percent time in SWS compared to the other two groups, and this persisted two weeks after defeat. **C:** Percent time spent in REM sleep during the light period in control (n = 7), passively (n = 15), and actively (n = 8) coping rats. Defeated groups displayed a higher percentage of their time spent in REM compared to the control group at all timepoints, including baseline. Passive and active coping rats did not significantly differ in the percent of time they spent in REM sleep at any time point.

*P<0.05, **P<0.01

**Figure 2. Percent Wake, SWS, and REM in the dark period at Baseline, Day 1, 4, and 7 of Defeat, and 2 weeks later**

**A:** Percent time spent awake during the dark period in control (n = 7), passively (n = 15), and actively (n = 8) coping rats. Groups did not significantly differ in the percent of time they spent in wake at any time point. **B:** Percent time spent in slow wave sleep (SWS) during the dark period in control (n = 7), passively (n = 15), and actively (n = 8) coping rats. Groups did not significantly differ in the percent of time they spent in SWS at any time point. **C:** Percent time spent in REM sleep during the dark period in control (n = 7), passively (n = 15), and actively (n = 8) coping rats. Defeated groups displayed significantly higher percentages of REM sleep than control rats at every time point, including baseline.

*P<0.05
