# Supplementary figures and images for: Passive Coping Strategies During Repeated Social Defeat Are Associated With Long-Lasting Changes in Sleep in Rats
Source: Front Syst Neurosci. 2020 Feb 19;14:6. doi: 10.3389/fnsys.2020.00006 (PMC7043017; doi:10.3389/fnsys.2020.00006)

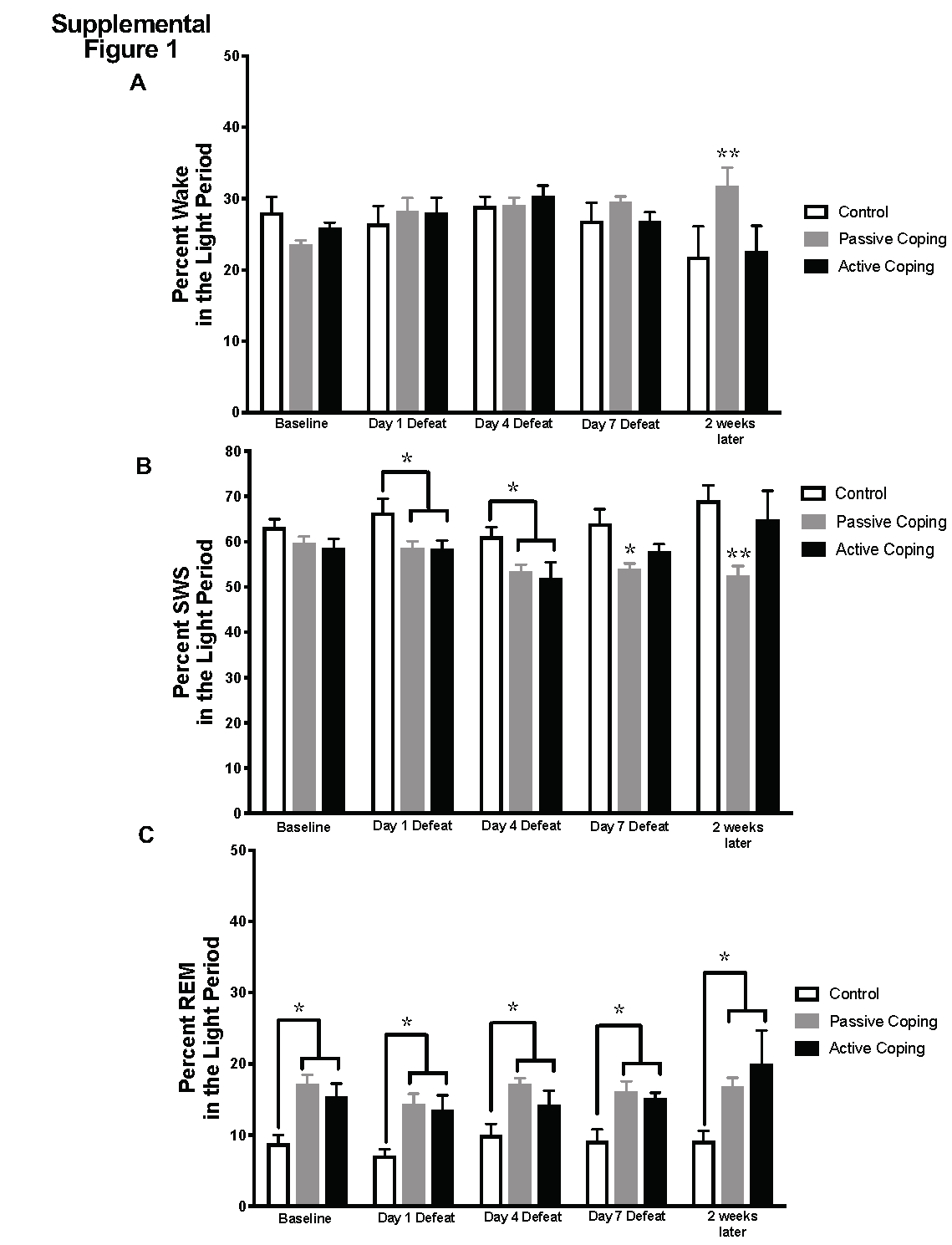

Supplement: Supplementary file 2 [file Image_1.tiff]

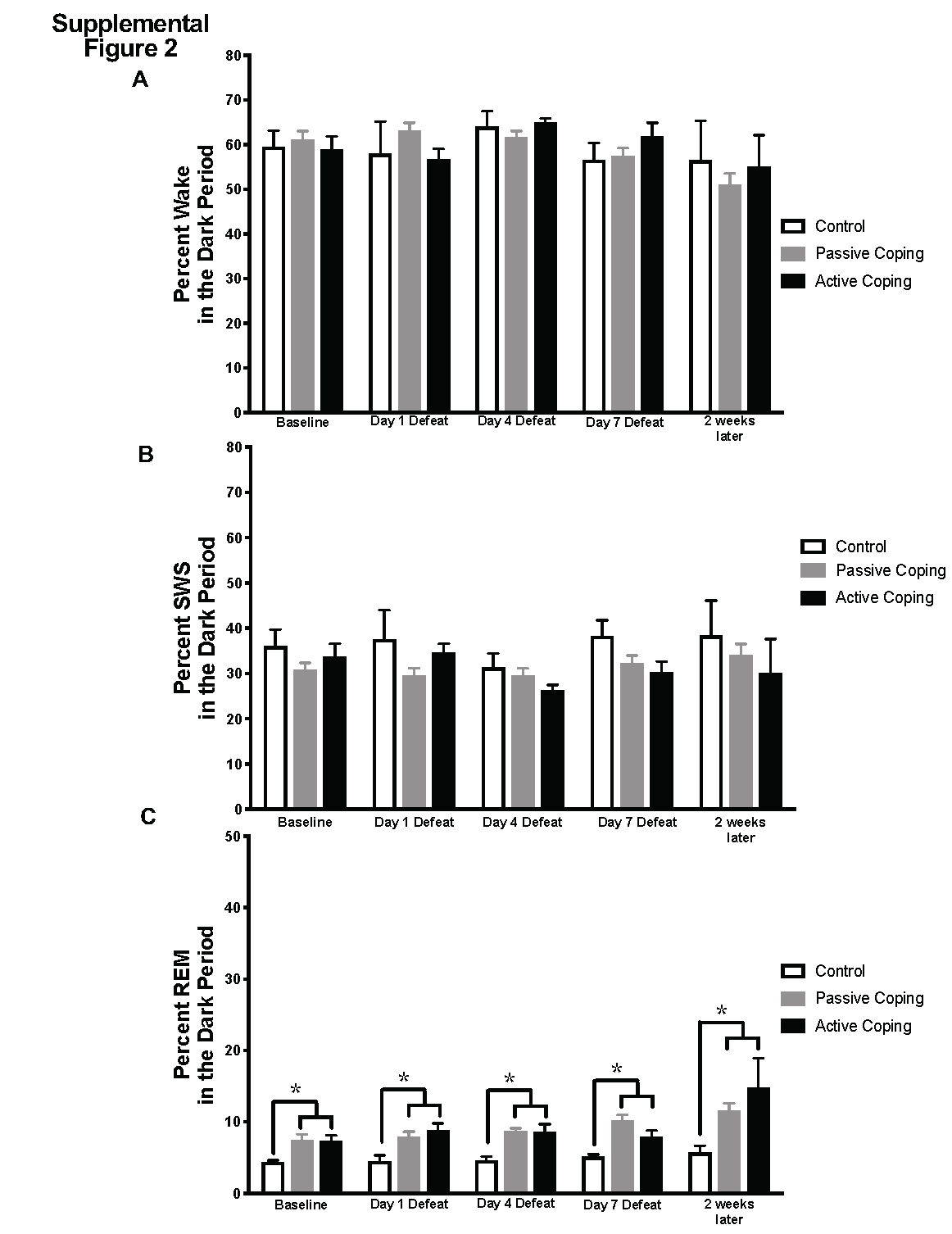

Supplement: Supplementary file 3 [file Image_2.tiff]
